# Supplementary material for: MetaRibo-Seq measures translation in microbiomes
Source: Nat Commun. 2020 Jun 29;11:3268. doi: 10.1038/s41467-020-17081-z (PMC7324362; doi:10.1038/s41467-020-17081-z)
Supplement: Supplementary file 10 — Supplementary Data 7 [file 41467_2020_17081_MOESM10_ESM.zip › File2/Confidence_High_Taxonomy/94612_out.krona.html]

Javascript must be enabled to view this page.

members
magnitude
magnitudeUnassigned
count
unassigned
taxon
rank

94612\_out

2

2
superkingdom
2759

kingdom
4751
2

2
451864
subkingdom

2
phylum

SRS051031\_contig\_number\_593
4890
1

1
subphylum
147538

1
147550
class

1
subclass
222543

1
5125
order

1
family
110618

genus

SRS1041145\_contig\_number\_31421
40628
1
